# Comment on “MSCohi-O lenses for long-term retention of mesenchymal stem cells on ocular surface as a therapeutic approach for chronic ocular graft-versus-host disease”

**DOI:** 10.1016/j.stemcr.2025.102401

**Published:** 2025-02-06

**Authors:** Xinxin Yu, Shuai Huang, Yizhuo Zhao, Aijun Deng, Lusheng Ma

**Affiliations:** 1 School of Clinical Medicine, Shandong Second Medical University, Weifang, China; 2 Binzhou Medical College, Yantai, China; 3 Department of Ophthalmology, Yantai Yuhuangding Hospital, Yantai, China

## Main text

We have recently read the paper titled “MSCohi-O lenses for long-term retention of mesenchymal stem cells on ocular surface as a therapeutic approach for chronic ocular graft-versus-host disease” (Volume 18, Issue 12, pp. 2356–2369, December 12, 2023), authored by Yuanyue Liu et al. and published in Stem Cell Reports. This study holds substantial importance in investigating dry eye associated with graft-versus-host disease. Following a thorough paper analysis, we have identified several questions regarding its content and are keen to engage in dialogue with the authors and other relevant stakeholders. In this study, the researchers employed MSCohi-O lenses with varying concentrations of UCMSCs in a New Zealand rabbit alkali burn model, replacing the lens with a new one every four days. While this experimental design appears to be well-founded, there are certain data and outcomes that the authors did not address, which are of particular interest to us. Consequently, we would like to pose the following questions. (1) What is the survival rate of UCMSCs on MSCohi-O lenses over time? Specifically, how many cells survive by day 4, and how many by day 7? (2) What is the rationale for replacing the MSCohi-O lens on day 4? What considerations informed the selection of this specific time point?

Secondly, it is noteworthy that the authors of this paper have employed the alkali burn model rather than the oGVHD model. The existing literature predominantly utilizes the classic allogeneic bone marrow transplantation model (Perez et al., 2016; Steven et al., 2023) or the humanized mouse model (Adhikary et al., 2021) to investigate ocular alterations. In contrast, this study adopts the rat alkali burn model to explore ocular graft-versus-host disease, raising the question of its broader acceptance in the field. Could the alkali burn model serve as a viable alternative to the traditional graft-versus-host disease models? Renaming the paper to “Ocular graft-versus-host disease” could significantly benefit ophthalmology researchers focused on this condition. This development would be particularly advantageous for ophthalmologists engaged in graft-versus-host disease research, and we are keenly interested in a favorable outcome to this inquiry. We fully acknowledge the complexity and challenges inherent in scientific research and hold the authors in high regard. We are reaching out with these inquiries to contribute to and expand upon the existing research in this field through meaningful dialogue. We look forward to the journal’s support in facilitating this exchange and our collaborative exploration of these issues.

## Declaration of interests

The authors declare no competing interests.
